# Evaluating Student Attitudes: Perceptions of Interprofessional Experiences Following Participation in a Student-Run Free Clinic

**DOI:** 10.7759/cureus.1053

**Published:** 2017-02-23

**Authors:** Aleksandr Kovalskiy, Rahim Ismail, Kelvin Tran, Anand Desai, Amna Imran, Caridad Hernandez

**Affiliations:** 1 College of Medicine, University of Central Florida

**Keywords:** student-run free clinic, medical students, pharmacy, interdisciplinary education

## Abstract

It is increasingly evident that patient health outcomes are improved when they are treated by an effective interdisciplinary healthcare team. Many also endorse that learning to function collaboratively in interdisciplinary settings should start at the onset of one’s medical education. Student-run free clinics, such as the University of Central Florida College of Medicine’s (UCF COM) KNIGHTS (Keeping Neighbors In Good Health Through Service) Clinic, provide opportunities for students to work in concert with other healthcare professionals. This study aimed to discern whether volunteering in this setting had a positive impact on medical students’ perception of working within an interdisciplinary team. A single survey was distributed via Qualtrics to all first and second-year medical students (N = 248) at the UCF COM. The items of interest examined in this study were part of a larger study described elsewhere. The mean responses on a 5-point Likert-like scale to these survey items were recorded and compared between two cohorts: KNIGHTS volunteers and non-volunteers. One hundred twenty-three (49.6%) students responded to the survey and most items showed no statistically significant difference between the two groups (p-value > 0.05). However, there were a few items of interest that did show a significant difference. These included KNIGHTS volunteers being much more likely to have worked with other healthcare professionals (p < 0.001) as well as believing themselves to have a better understanding of the role of medicine within an interprofessional team (p = 0.016). Additionally, KNIGHTS volunteers were more likely to feel like they understood the role of patient education (p = 0.031) and pharmacy (p = 0.040) within an interprofessional team. Interestingly, KNIGHTS volunteers were also more likely to believe that problem-solving skills should be learned with students within their own discipline (p = 0.009) as well as that there is little overlap between the roles of medical students and students from other healthcare disciplines (p = 0.044). Still, overall results showed that both volunteers and non-volunteers had an overall positive perception of interdisciplinary teams and working with other healthcare professionals.

## Introduction

Contemporary models of care are emphasizing the adoption of interprofessional team approaches to healthcare delivery. Many hospitals and practice entities are calling for healthcare professionals from multiple disciplines (i.e. physicians, nurses, pharmacists, physician assistants, etc) to work collaboratively in the care of increasingly complex patient populations. Moreover, a number of international, national, and regulatory agencies and organizations are advocating for the increased use of interprofessional healthcare approaches. For example, the World Health Organization (WHO), in a report on interprofessional education and collaborative practice, stated that “Interprofessional health-care teams understand how to optimize the skills of their members, share case management and provide better health-services to patients and the community” and that “the resulting strengthened health system leads to improved health outcomes” [1]. In the same report, the WHO found that when treated by an interprofessional healthcare team, patients reported better compliance and were overall more satisfied with their care [1]. These statements were echoed by Reeves, et al. who found that interprofessional healthcare teams contributed to increased patient outcomes, satisfaction, and adherence rates [2]. Dr. Darrell Kirch, president of the Association of American Medical Colleges (AAMC), has written that “interprofessional teams in health care are showing promise in achieving the triple aim - providing better care for the individual patient, reducing costs, and improving population health [3].” Closer to the Central Florida area, a study performed at a student-run free clinic in Jacksonville, Florida, also found that health issues that can be overlooked by other healthcare models are better identified and corrected by an interprofessional model [4]. Additionally, studies have shown that students in a variety of professional schools have increased self-reported team skills, long-term condition management abilities, and overall confidence after participating in collaborative interprofessional programs [5].

In order for effective interprofessional team collaborations to become the norm in healthcare, exposure to cross-professional learning opportunities should ideally occur early and throughout a health professional's training. Thus, opportunities for these experiences should be provided for learners in a wide range of healthcare professional schools. Many medical schools are incorporating such interprofessional experiences in their curriculums and exposing medical students to other healthcare professionals in their pre-clinical years [6]. Furthermore, given that an increasing amount of medical schools are affiliated with an interprofessional student-run free clinic, this can allow for integration and interaction between various healthcare students [6]. The University of Central Florida College of Medicine (UCF COM) is one of the medical schools affiliated with a student-run free clinic: the Keeping Neighbors In Good Health Through Service (KNIGHTS) Clinic funded by the Diebel Legacy Fund at the Central Florida Foundation. This clinic provides free and reduced-fee longitudinal medical care to those that cannot afford traditional healthcare; students volunteering at this clinic work with attendings and other students from medicine, pharmacy, and social work to help provide this care to the patients. A small number of medical schools even offer an elective course that integrates volunteering in a student-run free clinic along with periodic lectures and presentations, as well as required student reflections on their experiences [6-7]. A few of these schools, including the Medical University of South Carolina (MUSC) and the University of Minnesota Medical School, have surveyed students about their perceptions and attitudes about various aspects of healthcare, including working with other healthcare professionals, after volunteering in a student-run free clinic. For example, a number of these studies have shown that, after volunteering in an interprofessional student-run free clinic, positive student attitudes towards working in these types of healthcare teams either increased or were maintained [6-8]. It is these types of attitudes that can and should translate in the future into better patient compliance and outcomes.

There have only been a limited number of studies that have evaluated the perception of healthcare students working with other healthcare professionals [6-8]. The purpose of this study is to use both validated and self-made questionnaires to determine whether volunteering at the KNIGHTS clinic had a positive impact on medical students’ perceptions of working within an interdisciplinary team. It is our hypothesis that, like at the MUSC and the University of Minnesota Medical School, the students participating in the KNIGHTS clinic will also experience a greater appreciation and understanding towards other members of the healthcare team after volunteering at the clinic. It is our hope that after evaluating and presenting our results, more students will be encouraged to participate in the KNIGHTS clinic and, more broadly, more medical schools and their students will establish and/or participate in a student-run free clinic of their own. Furthermore, this study, along with previously completed studies in interprofessional healthcare, may serve as the groundwork for other longitudinal studies that go beyond perceptions and attitudes only in medical school and continue into a student’s residency training and practice.

## Materials and methods

During the fall semester of 2015, we conducted an online confidential survey (consisting of 30 items) of all first (M1) and second-year (M2) medical students at the UCF COM which emulated the MUSC (Shrader, et al.) study. Our main method of data collection was via survey responses using Qualtrics (Provo, Utah) software (for which the UCF COM owns a license) [9]. Potential participants were recruited via email through the UCF COM Office of Assessment, in-person by a research member before or after a class session, and via Facebook. Participation in this study was entirely voluntary and participants were free to withdraw at any point without penalty. Additionally, all students who completed the survey were compensated twenty dollars. The preliminary analysis of the collected data was completed in October 2015.

The administered survey was broken into four sections. This particular research study only evaluated the responses to one of the four sections, and the pertinent questions are shown in the results section of this text as well as in the appendix. The other three sections will be analyzed in other research studies. The section of interest contains demographic questions as well as sixteen questions that assess interdisciplinary attitudes and perceptions. This section of the survey was principally adopted from the Shrader, et al. MUSC study that was mentioned in the introduction and changed minimally. Only one question from this survey was modified to meet the needs of the current study: the original item read “I understand the respective role of physical therapy within an interprofessional team” and was modified to “… respective role of patient education within … ” in the current survey as KNIGHTS volunteers are not exposed to physical therapists at the clinic. The original survey is available in Shrader, et al. As part of the MUSC study, the researchers used survey questions taken from a questionnaire developed by Parsell and Bligh to assess the readiness of health care students for interprofessional learning (RIPLS) [10]. The survey items taken from the RIPLS questionnaire are indicated in the results section of this text.

Any M1 or M2 UCF College of Medicine medical student was eligible to participate in this research study regardless of whether or not they had ever volunteered at the KNIGHTS Clinic. The collected data was analyzed and compared between all KNIGHTS Clinic volunteers (KNIGHTS volunteers) and those who have not volunteered (non-volunteers). The non-volunteers cohort served as our control group and the KNIGHTS volunteers cohort served as our experimental “exposed” group.

Prior to partaking in the study, all potential participants were provided with an informed consent page at the beginning of the survey. At that time, any potential participant either chose to consent or not consent to the research study parameters, before they took the survey. In order to maintain the confidentiality and privacy of all study participants, all data was obtained and de-identified by the Office of Assessment prior to statistical analysis of the results and the Office of Assessment alone had access to the names of the participants. Once all of the survey responses were generated, the data was downloaded from the Qualtrics software and encrypted and stored on password-protected computers. The recruitment methods, data storage, and data analysis techniques have all been approved by the Institutional Review Board at UCF.

### Statistical analysis

There are two cohorts whose results were collected and analyzed in this study: those students who volunteered at the KNIGHTS clinic and those who did not volunteer. Categorical variables were reported as frequencies and percentages while ordinal variables were presented as means and standard deviations. Demographic data were summarized using descriptive statistics. An independent samples t-test was used to analyze between-group comparisons. All tests are two-sided and p-values < 0.05 are considered statistically significant. Statistical analyses were conducted using SPSS 22.0 (IBM, NY, USA) [11].

## Results

Two hundred forty-eight (248) medical students were surveyed and a total of 136 students (M1s = 53 and M2s = 73) completed the survey; however, 13 of these responses were incomplete and were missing data for the variables/items of interest. As such, these students’ data was deleted and 123 of the 136 responses were analyzed (49.6% corrected response rate). The demographics of the students who responded are shown in Table 1.

**Table 1:** Description of survey volunteer demographics [i] All data except age reported as frequency and percentage [ii] Reported as mean and standard deviation [iii] Reported as frequency and percentage of volunteers and non-volunteers who HAVE had PRIOR volunteer experiences

Description of survey volunteer demographics			
	KNIGHTS Volunteers	Non-Volunteers	Total
Number of Participants [i]	50 (40.7%)	73 (59.3%)	123
1st year student	3 (6.0%)	50 (68.5%)	53 (43.1%)
2nd year student	47 (94.0%)	23 (31.5%)	70 (56.9%)
Male	26 (52.0%)	31 (42.5%)	57 (46.3%)
Female	24 (48.0%)	41 (56.2%)	65 (52.8%)
Other	0 (0.0%)	1 (1.3%)	1 (0.8%)
Asian/Pacific Islander	13 (26.0%)	26 (35.6%)	39 (31.7%)
Hispanic	3 (6.0%)	3 (4.1%)	6 (4.9%)
White	28 (56.0%)	41 (56.2%)	69 (56.1%)
Other	6 (12.0%)	3 (4.1%)	9 (7.3%)
Age (Mean) [ii]	24.94 (2.817)	24.39 (3.284)	24.62 (3.099)
Prior volunteer experience [iii]	44 (88.0%)	65 (89.0%)	109 (88.6%)

Of the 123 responses, most were from non-volunteers (59.3% vs 40.7%), and most were second-year students (56.9% vs 43.1%). Male students accounted for 46.3%, female students 52.8% (with 0.8% identifying as “other”), and 56.1% identified as white. Additionally, the vast majority of responders have had some type of prior volunteer experience (88.6%). For question 13-7 (My previous volunteer clinical experiences has positively influenced my attitude towards working in an interdisciplinary team), of the 108 responses, the vast majority either agreed or strongly agreed (75.6%) that their prior volunteer experiences positively influenced their current attitudes towards working in interdisciplinary teams. Of the 50 KNIGHTS clinic volunteers who answered question 21-7 (The KNIGHTS Clinic positively influenced my attitude towards working in an interdisciplinary team), 94.0% of them either agreed or strongly agreed with the statement. The results for items 13-7 and 21-7 are shown in Table 2.

**Table 2:** Survey volunteers’ current attitudes towards working in interdisciplinary teams [i] 15 students did not answer this question and percentages are reported as the total number of students who took the survey as a whole [ii] This question applied to KNIGHTS Clinic volunteers only and frequencies and percentages reported are based on the 50 KNIGHTS volunteers

Survey volunteers’ current attitudes towards working in interdisciplinary teams							
	Strongly Disagree	Disagree	Neutral	Agree	Strongly Agree	Not Applicable	Total
Question 13-7 (influence of previous volunteering experiences) [i]	1 (0.8%)	2 (1.6%)	10 (8.1%)	38 (30.9%)	55 (44.7%)	2 (1.6%)	108
Question 21-7 (KNIGHTS Clinic influence on attitudes) [ii]	0	0	3 (6.0%)	17 (34.0%)	30 (60.0%)	0	50

The survey items of interest and the mean responses on a Likert-like scale (with '1' being strongly disagree and '5' being strongly agree) are displayed in Table 3.

**Table 3:** Mean and independent sample t-test analysis of student responses to question 24 [i] Statistically significant p-values (< 0.05) are bolded and italicized [ii] Question items 1 through 8 were taken from the MUSC Shrader, et al. study [iii] This item was modified from “ … role of physical therapy… ” to “ … role of patient education … ” [iv] Question items 10 through 17 were taken from the RIPLS questionnaire developed by Parsell and Bligh [v] Question 24 items 10 and 12 were reverse scored (1 being strongly agree, 2 being agree, etc) such that lower scores can be interpreted similarly to the other items in the survey

Mean and independent sample t-test analysis of student responses to question 24			
Question 24	KNIGHTS Volunteer (mean)	Non-volunteer (mean)	p-value [i]
*I have worked with students from other health professions in an interprofessional team. (1) [ii]*	*4.54*	*3.48*	*<0.001*
I am confident in my abilities to effectively work within an interprofessional healthcare team to develop a realistic and appropriate patient care plan. (2)	4.26	4.14	0.352
*I understand the respective role of medicine within an interprofessional team. (3)*	*4.34*	*4.03*	*0.016*
*I understand the respective role of patient education within an interprofessional team. (4) [iii]*	*4.38*	*4.11*	*0.031*
*I understand the respective role of pharmacy within an interprofessional team. (5)*	*4.20*	*3.90*	*0.040*
It is important to interact with teachers and preceptors from other healthcare professions. (6)	4.36	4.44	0.522
Using interprofessional teams to deliver quality healthcare is essential for the future. (7)	4.50	4.56	0.572
I am going to work in an environment that fosters interprofessional teamwork to deliver patient care in the future. (8)	4.40	4.44	0.755
*Clinical problem-solving skills should only be learned with students from my own discipline. (10) [iv, v]*	*3.70*	*4.22*	*0.009*
I have to acquire more knowledge and skills than other students in other healthcare disciplines. (11)	3.35	3.58	0.228
*There is little overlap between my role and that of other students belonging to other healthcare disciplines. (12) [v]*	*3.65*	*4.05*	*0.044*
Shared learning and working within an interprofessional team will help me communicate better with patients and healthcare professionals. (13)	4.42	4.45	0.783
Shared learning and working within an interprofessional team will increase my ability to understand clinical problems. (14)	4.32	4.48	0.230
Shared learning and working within an interprofessional team will help me be a more effective member of a healthcare team in the future. (15)	4.42	4.56	0.204
Shared learning and working within an interprofessional team will help me understand my own limitations. (16)	4.44	4.45	0.914
Patients ultimately benefit if students and healthcare professionals work in interprofessional teams to solve patient problems. (17)	4.56	4.59	0.778

**Figure 1:**
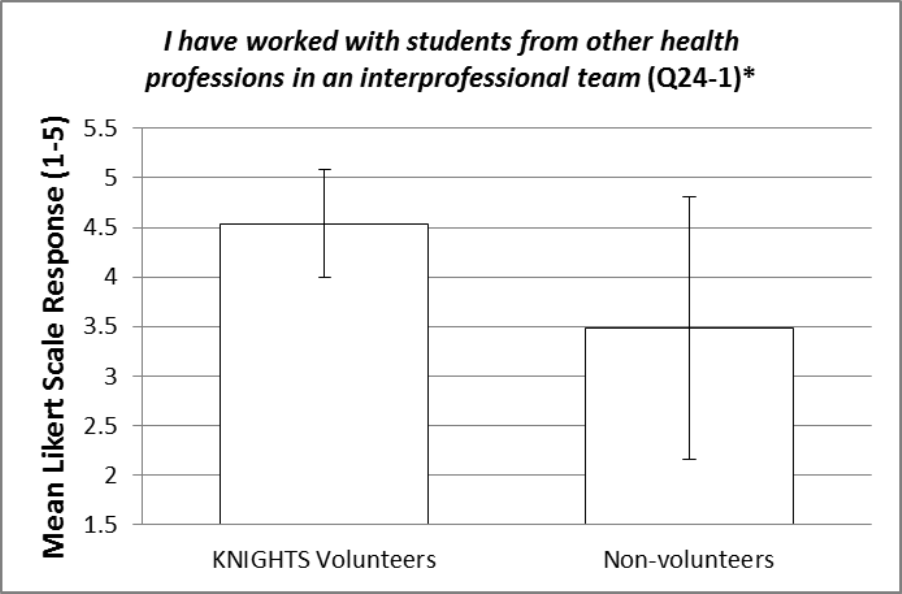
I have worked with students from other health professions in an interprofessional team (Q24-1) [*] p-value is statistically significant (< 0.001)

**Figure 2:**
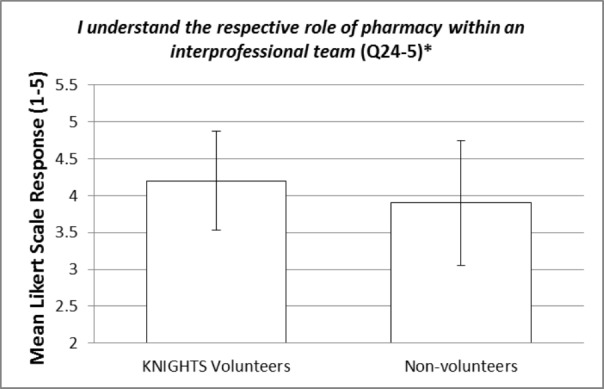
I understand the respective role of pharmacy within an interprofessional team (Q24-5) [*] p-value is statistically significant (0.040)

**Figure 3:**
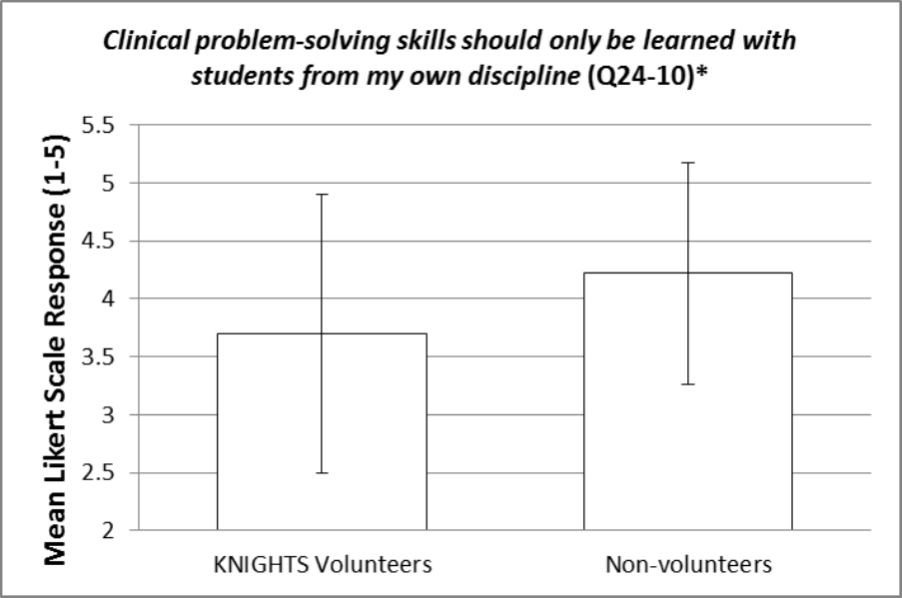
Clinical problem-solving skills should only be learned with students from my own discipline (Q24-10) [*] p-value is statistically significant (0.009)

**Figure 4:**
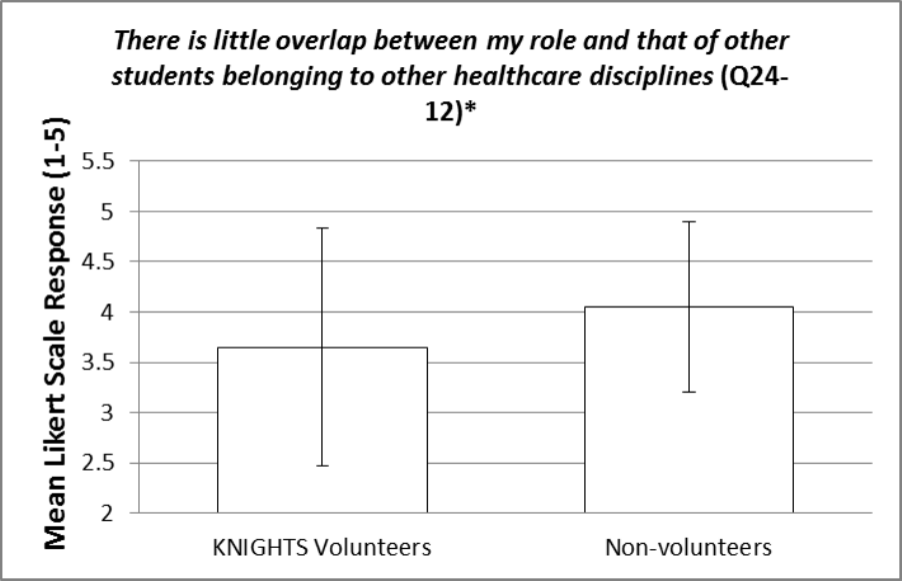
There is little overlap between my role and that of other students belonging to other healthcare disciplines (Q24-12) [*] p-value is statistically significant (0.044)

These items were compared between KNIGHTS Clinic volunteers and non-volunteers using an independent samples t-test. The statistically significant p-values (< 0.05) for the items within question 24 are bolded and italicized within Table 3. Items 24-10 and 24-12 are reverse-scored as described in footnote [v]. Most of the items did not show a statistically significant difference between KNIGHTS volunteers and non-volunteers. However, there were a few items of interest that were statistically significant and will be elaborated on in the discussion section. Additionally, representative figures of these items of interest can be visualized in Figures 1-4. Figures 1-4 represent the mean responses of students based on their cohort (volunteers vs non-volunteers) to select questions on the Likert-like scale of question 24. The error bars represent the standard deviation from the mean for the respective responses.

## Discussion

This study aimed to compare the attitudes and perceptions of first and second-year UCF COM students on working with other healthcare professions in interdisciplinary teams, based on whether or not they have volunteered at the student-run KNIGHTS clinic.

Some of our findings differed from the findings seen in the previous literature. Notably, responses to two statements showed significance: “Clinical problem-solving skills should only be learned with students from my own discipline” (volunteers agreed more) and “There is little overlap between my role and that of other students belonging to other healthcare disciplines” (volunteers agreed more strongly). In previous literature, these statements could not be proven significantly different between both groups. A further understanding of the KNIGHTS Clinic may elucidate these findings, however. For example, there is a close interaction between medicine and pharmacy in the KNIGHTS Clinic. Indeed, our survey found volunteers at KNIGHTS Clinic reported a significantly better understanding of the role of pharmacist than those who did not volunteer. This better understanding may have led to the belief that there is a difference in the respective education models, which is inherently true. In other words, they may believe that these different professions should learn separately but work collaboratively. Additionally, better understanding of said roles may also contribute to how volunteers answered in regards to the overlap of roles. Fundamentally, there are differences between the roles of physicians and other healthcare professions such as pharmacy so these results are not all that surprising. The KNIGHTS volunteers, having worked with pharmacy students, may have a better understanding of these differences in the roles of physicians/medical students and pharmacists/pharmacy students than the non-volunteer cohort; they may think that the intricacies of patient needs make it necessary for specialization and separation of healthcare professions.

A comparison of the majority of the other items was very promising. In fact, for both volunteers and non-volunteers, the responses were all indicative of positive perceptions of other healthcare professions and working in interprofessional teams. As alluded to earlier, our results also show that KNIGHTS Clinic volunteers consider themselves to have a better understanding of the role of the pharmacy profession in an interprofessional team than do non-volunteers. This is very important, especially in today’s medical setting; now, more than ever, physicians are increasingly working and interacting with pharmacists. If there is a disconnect or if there is an unclear understanding of respective roles, patient satisfaction and, more importantly, their health may suffer [12-13].

Also of note, both volunteers and non-volunteers reported that patients benefit when healthcare professionals work collaboratively in teams to solve patient problems, as represented by the high means (4.56 and 4.59, respectively) in response to the statement “Patients ultimately benefit if students and healthcare professionals work in interprofessional teams to solve patient problems”. These types of responses are expected of medical students as many people who enter medical school today are of similar mindsets when it comes to healthcare; they most likely agree that medicine should be practiced as a team and patient outcomes would benefit if done so. Additionally, the culture and opportunities provided by the UCF COM itself may also play a role in students’ beliefs. At the UCF COM, students participate in interdisciplinary seminars and experiences with other professions such as pharmacy, nursing, and social work. Having exposure to these in-class sessions most likely has a positive impact on how the medical students view other healthcare professions.

Some of the recognizable limitations of this study are that a within-groups comparison was not performed as was done in the Shrader, et al. study from which our survey tool was based; there was no pre- and post-clinic exposure comparison [6]. This may have provided more significant evidence to the direct effects of volunteering in such student-run free clinics. Additionally, other studies have shown that weighted contact hours, not simply volunteering or not, are a better basis for comparison between groups of volunteers, though not necessarily for the same parameters of interest as this study [14]. Using weighted contact hours may display that the more hours or times a volunteer is exposed to another healthcare professional, the more their attitudes and perceptions may change. This study only compared whether a student volunteered at least once or had not volunteered at all.

## Conclusions

In conclusion, our results are in overall agreement with the opinions and studies mentioned in the introduction: medical students’ participation in a student-run free clinic is beneficial to their attitudes and perceptions of working in interdisciplinary healthcare teams. Though our results are, for the most part, comparable to those of previous findings, further research should be performed in this field, taking the aforementioned limitations into account, in order to more fully substantiate ours and others’ data; these studies could also add measures to evaluate a true improved understanding of roles, rather than just a perceived increase. It is our hope that this research will be replicated by future students at the UCF COM so as to maintain a database of results and hopefully use this data to help further incorporate interprofessional learning and experiences such as the KNIGHTS Clinic into the UCF COM curriculum and the curriculums of medical schools across the United States. Further research may also help elucidate the true reasons for the unexpected results to the items asking if clinical problem-solving skills should only be learned with students from my own discipline (item 24-10) and if there is little overlap between my role and that of other students belonging to other healthcare disciplines (item 24-12). Most importantly (and quite encouragingly), regardless of volunteer experience, the medical students at UCF COM seem to appreciate the value of working in interdisciplinary teams and agreed that they improved patient outcomes. Such findings support the need for continued implementation of interdisciplinary interactions in medical education and beyond.
